# SRSF9 Regulates Cassette Exon Splicing of Caspase-2 by Interacting with Its Downstream Exon

**DOI:** 10.3390/cells10030679

**Published:** 2021-03-19

**Authors:** Jiyeon Ha, Hana Jang, Namjeong Choi, Jagyeong Oh, Chanhyuk Min, Davide Pradella, Da-Woon Jung, Darren R. Williams, Daeho Park, Claudia Ghigna, Xuexiu Zheng, Haihong Shen

**Affiliations:** 1 School of life Sciences, Gwangju Institute of Science and Technology, Gwangju 500-712, Korea; hajiyn@gist.ac.kr (J.H.); hana.jang@kitox.re.kr (H.J.); njchoi@gist.ac.kr (N.C.); jgoh@gist.ac.kr (J.O.); alscksgur@gist.ac.kr (C.M.); jung@gist.ac.kr (D.-W.J.); darren@gist.ac.kr (D.R.W.); daehopark@gist.ac.kr (D.P.); 2 Institute of Molecular Genetics “Luigi Luca Cavalli-Sforza”, National Research Council, Via Abbiategrasso 207, 27100 Pavia, Italy; davide.pradella@igm.cnr.it (D.P.); arneri@igm.cnr.it (C.G.)

**Keywords:** alternative splicing, SRSF9, caspase 2

## Abstract

Alternative splicing (AS) is an important posttranscriptional regulatory process. Damaged or unnecessary cells need to be removed though apoptosis to maintain physiological processes. Caspase-2 pre-mRNA produces pro-apoptotic long mRNA and anti-apoptotic short mRNA isoforms through AS. How AS of Caspase-2 is regulated remains unclear. In the present study, we identified a novel regulatory protein SRSF9 for AS of Caspase-2 cassette exon 9. Knock-down (KD) of SRSF9 increased inclusion of cassette exon and on the other hand, overexpression of SRSF9 decreased inclusion of this exon. Deletion mutagenesis demonstrated that exon 9, parts of intron 9, exon 8 and exon 10 were not required for the role of SRSF9 in Caspase-2 AS. However, deletion and substitution mutation analysis revealed that AGGAG sequence located at exon 10 provided functional target for SRSF9. In addition, RNA-pulldown mediated immunoblotting analysis showed that SRSF9 interacted with this sequence. Gene ontology analysis of RNA-seq from SRSF9 KD cells demonstrates that SRSF9 could regulate AS of a subset of apoptosis related genes. Collectively, our results reveal a basis for regulation of Caspase-2 AS.

## 1. Introduction

Splicing is an essential process in gene expression of higher eukaryotes, in which introns are removed from pre-mRNA to produce mature RNA (mRNA) [1,2]. Splicing requires multiple RNA-RNA and RNA-protein interactions to identify splice-sites and join them [3]. The splicing reaction occurs in a dynamic and large ribonucleoprotein complex called spliceosome [1,2]. Spliceosome contains small nuclear ribonucleoproteins (snRNPs) such as U1, U2, U4, U5, U6 snRNP, their associated proteins and other non-snRNP protein factors [4]. In stepwise assembly of spliceosome, prespliceosome (complex A) is formed first, subsequently producing catalytically active spliceosome (complexes B and C) [2]. Alternative splicing (AS) is a mechanism by which eukaryotic cells generate multiple mRNA isoforms with different functions from a single gene to increase gene diversities [5,6,7]. AS occurs either in open reading frames to affect functions of the encoded protein or regulatory sequences in untranslated regions (UTRs) to influence RNA stability, transport and translation [7]. AS events mainly include exon skipping (SE), alternative 5′ and 3′ splice site selection (A5SS and A3SS), intron retention (RI) and mutually exclusive exons (MXE). More than 95% of human genes produce more than one mRNAs by AS. Thus, defects of AS can result in various human diseases including cancer and neurodegenerative diseases [3,8,9,10,11].

**Figure 1:**
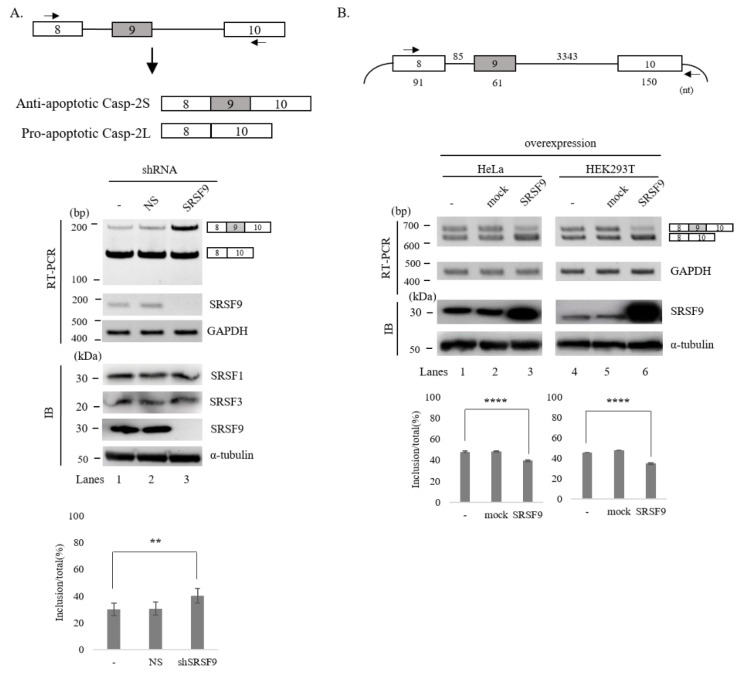
SRSF9 regulates AS of Caspase-2 pre-mRNA. (**A**)-(Upper) Schematic AS representation of endogenous Caspase-2 pre-mRNA. Primers used in RT-PCR are shown with arrows. (Middle) RT-PCR analysis of endogenous Caspase-2 pre-mRNA from untreated, ns- and SRSF9 shRNA treated cells. GAPDH was used as a loading control. Results of immunoblotting with anti-SRSF9 in these cells are shown, with α-tubulin, SRSF1 and SRSF3 as a controls. (Lower) Statistical differences of RT-PCR with *p* values are shown, ****, *p* < 0.0001 and **, *p* < 0.01. (**B**)-(Upper) Schematic representation of Caspase-2 minigene. Exons are shown with boxes and introns are shown with lines. Lengths of each part are shown. Primers used in RT-PCR analysis are shown with arrows. (Middle) RT-PCR analysis of Caspase-2 minigene from untreated, empty vector treated and SRSF9 expression plasmid treated HeLa and HEK293T cells. GAPDH was used as a loading control. Immunoblotting with anti-SRSF9 antibody is shown, with α-tubulin as a control. (Lower) Statistical differences of RT-PCR analysis are shown, ****, *p* < 0.0001; and **, *p* < 0.01.

Apoptosis is a general way to remove damaged or unnecessary cells at specific physiological state [12]. Disrupted regulation of apoptotic signaling pathway is involved in various diseases. While excessive apoptosis can result in neurodegenerative diseases, insufficient apoptosis causes cancer and induces tumor cells to be resistant to therapeutics [12,13]. Interestingly, it has been observed that AS often results in proteins with opposite functions in apoptosis, such as Fas receptor (APO-1/CD95), Caspase-8, c-FLIP, Apaf-1, ICAD, Bcl-x, Mcl-1 and Bim [14,15,16,17,18,19,20,21,22]. Caspases are expressed in an inactive proenzyme form. They often activate other procaspases once activated [23]. Caspase-2 is the most evolutionarily conserved caspase [24]. It has roles in apoptosis initiating, tumor suppressing and cell response to DNA damage [25,26,27,28]. AS of Caspase-2 occurs in response to pro-apoptotic signals to generate two mRNA isoforms, a long a pro-apoptotic Caspase-2L resulting from exclusion of cassette exon and a short anti-apoptotic Caspase- 2S produced by inclusion of cassette exon that recruits a premature stop codon and a shorter protein (Figure 1A, Upper) [27]. While SRSF1, SRSF2, SRSF3, RBM5 and PTP inhibit the inclusion of cassette exon to produce Caspase-2L isoform, hnRNP A1 stimulates the inclusion of cassette exon and the synthesis of Caspase-2S [29,30,31,32,33].

SRSF9 is a member of Serine-Arginine rich (SR) protein family. SR proteins contain at least one RNA recognition motif (RRM) and RS domains. They play important roles in AS and constitutive splicing [34]. SR proteins have regulatory roles in constitutive and alternative splicing by facilitating the recruitment and assembly of splicing machinery through protein-protein or protein-RNA interactions [34]. In vitro systematic evolution of ligands by exponential enrichment (SELEX) analysis has demonstrated that SRSF9 prefers AGSAS (S = G or C) sequences for binding [35]. It has been demonstrated that SRSF9 can regulate 5′ splice-site selection, 3′ splice-site selection and exon skipping of SMN, Bcl-x, CD44 and hnRNP A1 pre-mRNA [36,37,38,39,40]. In addition to its roles in splicing, SRSF9 can also inhibit RNA editing and provide targets for microRNA to inhibit cell proliferation and induce apoptosis [41,42,43].

We have previously reported that SRSF3 can regulate AS of Caspase-2 by interacting with exon 8 [30]. In this manuscript, we identified a novel regulatory protein SRSF9 for AS of Caspase-2 cassette exon 9. While SRSF9 knocking down (KD) promoted inclusion of cassette exon, SRSF9 overexpression inhibited the inclusion of this exon. To locate functional targets of SRSF9 in AS of Caspase-2, we performed deletion and substitution mutagenesis. We found that exon 9, parts of intron 9, exon 8 and exon 10 were not required for the roles of SRSF9 roles in Caspase-2 AS. However, we demonstrated that AGGAG sequence at exon 10 was the functional target for SRSF9 in AS of Capase-2. Furthermore, SRSF9 interacted with this sequence based on RNA-pulldown and immunoblotting analysis. Notably, we performed gene ontology analysis with RNA-seq from SRSF9 KD cells and found that SRSF9 regulated AS of a number of apoptosis related genes. Our results provided a basis for the regulation of Caspase-2 AS.

## 2. Materials and Methods

### 2.1. Cell Culture, RNA Extraction and RT-PCR

HeLa and HEK293T cells were cultured in Dulbecco’s Modified Eagle’s Medium (DMEM) (HyClone, Marlborough, MA, USA, SH30243.01) medium supplemented with 10% fetal bovine serum (FBS, HyClone, SH30084.03), 2 mM glutamine, 100 U/mL penicillin and 100 μg streptomycin at 37 °C in a 5% CO_2_ incubator. Total RNAs were extracted from cells using Trizol reagent (MRC, London, UK, TR118) according to the manufacturer’s instruction. Reverse transcription was performed using M-MLV reverse transcriptase (Elpis, Daejeon, Korea, EBT-1028). These RNAs (1 μg) were then used to synthesize cDNAs followed by PCR amplification using 0.5 μg cDNA as template with GAPDH as an internal control. Primers used for RT-PCR analysis are provided in Supplementary Table S1.

### 2.2. Plasmid Construction

D9-1, D9-2, D9-3, D9-4, D-10, M1 and M2 plasmids were constructed by overlapping PCR in pCI-neo-caspase-2 [30] plasmid using primer pairs listed in Supplementary Table S1.

### 2.3. shRNA Treatment and Plasmid Transfection

SRSF9 shRNA viruses were produced by transfecting a mixture of SRSF9 shRNA plasmid, pSPAX2 and pMD2G plasmids into 293T cells using polyethyleneimine (PEI) reagent as previously described [44]. After 24 h incubation, the supernatant was collected after centrifuging at 5000 rpm for 3 min at 4 °C. The supernatant was used to infect cells for 72 h after adding 10 μg/mL polybrene. Plasmid transfection was performed using PEI reagent as previously described [44].

### 2.4. RNA Pulldown Assay and Immunoblotting Assay

RNA pulldown analysis was performed as previously described [30]. In brief, streptavidin agarose conjugate (Millipore, Burlington, MA, USA, 16-126) was used to covalently link biotin labeled RNA, followed by incubation with cellular extract at 4 °C for 4 h in buffer D (20 mM Tris–Cl, 300 mM KCl, 2 mM EDTA, 20% glycerol, 0.5 mM DTT, 0.5 mM PMSF, pH 7.5). Immunoblotting analysis was performed using anti-SRSF9 (Invitrogen, Carlsbad, CA, USA, MA5-26990), anti-SRSF1 (Santa Cruz Biotechnology, Dallas, TX, USA, SC-33652) and anti-SRSF3 antibody (Invitrogen, 334200) as previously described [30].

### 2.5. Splicing Analysis of RNA-seq and Gene Ontology (GO) Analysis

AS analysis RNA-seq data was performed using replicate multivariate analysis of transcript splicing (rMATS) [45]. GO analysis of enriched functions was performed using DAVID Bioinformatics Resources 6.8 https://david.ncifcrf.gov/ (accessed on 15 January 2021) [46].

### 2.6. Correlation Analysis

Casp-2 exon 9 inclusion (PSI; Percent Spliced-In) and SRSF9 expression levels correlation analysis in different human tissues was performed by using the Pearson’s correlation method. The Vertebrate Alternative Splicing and Transcription Database (VastDB). Available online: http://vastdb.crg.eu/ (accessed on 21 February 2021) [47] was used to retrieve human Casp-2 exon 9 PSI values and SRSF9 cRPKM (corrected Reads Per Kilobasepair and Million mapped reads) in 31 sets of tissues, including lymph nodes, bladder, thymus, endometrial stroma, prostate, small intestine, testis, spleen, ovary, stomach, melanocytes, adrenal, endothelium, placenta, breast, kidney, thyroid, white blood cells, bone marrow, brain, lung, skin, liver, colon, adipose tissue, pancreas, mononuclear cells and hearth. Tissues in which Casp-2 exon 9 event does not meet the minimum threshold for the quality score were excluded.

### 2.7. Annexin V/PI Staining

FITC Annexin V Apoptosis Detection Kit I (BD biosciences, 556547) was used to perform Annexin V/PI staining. 10^5^ of shRNA lentivirus treated HeLa cells were resuspended with 100 μL of 1X Annexin V binding buffer. The 3 μL of FITC-Annexin V and 2 μL of PI were added and followed by incubation for 15 min in ice. The population of apoptotic cells were analysed using flow cytometry.

### 2.8. Quantitation and Statistical Analysis

All experiments were performed in triplicate. Statistical differences among three groups were evaluated with one-way analysis of variance (ANOVA). Statistical significances were indicated by asterisk (****, *p* < 0.0001; ***, *p* < 0.001; **, *p* < 0.01; and *, *p* < 0.05)

## 3. Results

### 3.1. SRSF9 Regulates AS of Caspase-2

We have previously shown that SRSF3 can regulate AS of Caspase-2 through interacting with exon 8 [30]. To further understand regulatory mechanisms of Caspase-2 AS, we applied SpliceAid2 (http://www.introni.it/spliceaid (accessed on 23 October 2020)) [48] and RBPmap (http://rbpmap.technion.ac.il (accessed on 23 October 2020)) to predict possible binding proteins on Caspase-2 pre-mRNA [49]. We found that Caspase-2 pre-mRNA contained ~20 potential binding motifs for SRSF9 at different locations (Supplementary Table S2) (Supplementary Figure S1). To test the regulatory possibility of SRSF9 in the AS of Caspase-2, we performed KD HeLa cells using SRSF9-targeting lentivirus shRNA. As a control, non-silencing (NS) shRNA was used. As shown in Figure 1A (Lower), following SRSF9-targeting shRNA treatment, SRSF9 was hardly detectable by RT-PCR and immunoblotting using an anti-SRSF9 antibody (lane 3). By contrast, NS shRNA treatment did not cause a decrease in SRSF9 expression (lane 2). Notably, SRSF9 KD induced a significant increase of cassette exon (exon 9) inclusion of Caspase-2 (~11%, the cassette exon included RNA ratio in total RNA) (lane 3) and accordingly a significant decrease of exon skipping, while NS shRNA did not affect its AS (lane 2). As expected, reduced SRSF9 expression promoted endogenous exon 9 inclusion of Caspase-2. We next wondered whether increased SRSF9 might have opposite effects compared to SRSF9 KD. To this end, we applied a minigene produced in our laboratory previously [30], which contained genomic sequence from exon 8 through exon 10 (Figure 1B, Upper). As shown in Figure 1B (Lower), overexpression of SRSF9 resulted in significantly decreased inclusions of cassette exon in both HeLa and HEK293T cells (~12% and ~11% independently) (Lanes 3 and 6).

Finally, to explore the link between SRSF9 and Caspase-2 exon 9 AS in different human tissues and cell populations, we retrieved SRSF9 expression levels and *Caspase-2* exon 9 PSI (Percent Spliced-In) values among different human tissues annotated in the VastDB. Notably, we found a significant correlation (r = −0.4088; *p*-value = 0.0224) between *SRSF9* expression and Caspase-2 exon 9 inclusion, thus confirming that low levels of SRSF9 promote Caspase-2 exon 9 inclusion in the final mRNA (Supplementary Figure S2).

Collectively, SRSF9 KD and overexpression experiments and the SRSF9/Caspase-2 AS correlation analysis in different human tissues indicate that SRSF9 can regulate AS of Caspase-2. 

### 3.2. SRSF9 Targets Downstream Exon to Regulate AS of Caspase-2

**Figure 2:**
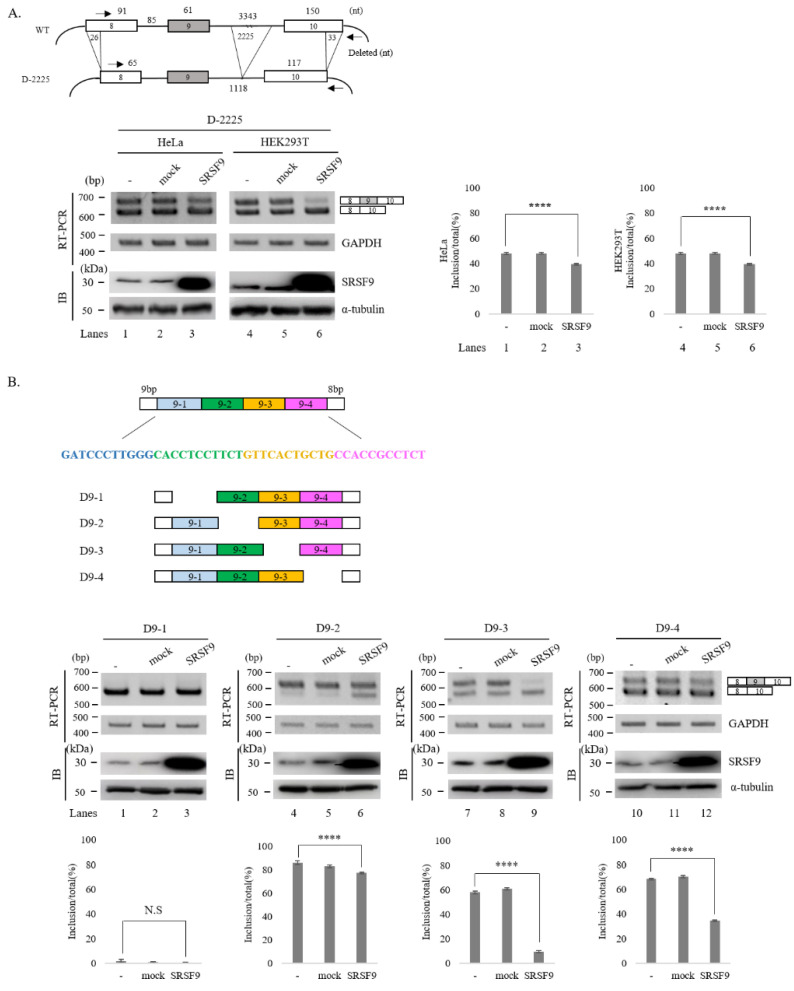
Some sequences of intron 9, exon 9, exon 8 and exon 10 are not required for SRSF9 function. (**A**)-(Upper) Schematic representation of D-2225 minigene. Lengths of deleted regions are shown. (Middle) RT-PCR analysis of D-2225 minigene in untreated, empty vector treated and SRSF9 expression plasmids treated HeLa and HEK293T cells. Statistical differences of RT-PCR analysis are shown in the right, ****, *p* < 0.0001. (**B**)-(Upper) Schematic representations of D9-1, D9-2, D9-3 and D9-4 minigenes. Deleted sequences in each minigene are shown. (Middle) RT-PCR analysis of D9-1, D9-2, D9-3 and D9-4 minigenes in untreated, empty vector treated and SRSF9 expression plasmids treated HeLa cells. (Lower) Statistical differences of RT-PCR analysis are shown, ****, *p* < 0.0001.

Because Caspase-2 pre-mRNA contains multiple potential SRSF9 binding sequences at different positions, we first applied deletion mutation to determine RNA sequences responsible for SRSF9 function in AS of Caspase-2. We first applied a deletion mutant that was used to locate functional target of SRSF3, in which 2225 nucleotides (nt) of intron 9, 26 nt of exon 8 and 33 nt of exon 10 were deleted (D-2225) (Figure 2, Upper) [30]. As shown in Figure 2A, Lower, in this mutant, overexpression of SRSF9 still increased cassette exon skipping with significant levels in HeLa and HEK293T cells independently (~10% and ~12% respectively). Thus, deleted RNA sequences in the D-2225 mutant minigene were not required for the SRSF9 function in Caspase-2 AS. We next wondered whether cassette exon 9 contained functional RNA sequences for SRSF9 in Caspase-2 AS. We produced four deletion mutants from D-2225 mutant in which 11 nt were deleted individually in each mutant (D9-1, D9-2, D9-3 and D9-4) (Figure 2B, Upper). As shown in Figure 1B (Lower), D9-1 generated skipped isoform primarily (lane 1). Thus, further effects of SRSF9 on exon skipping were not observable with this mutant (lane 3). Similar to the wild type minigene, other mutants (D9-2, D9-3 and D9-4) produced more cassette exon skipped isoforms by overexpressing SRSF9 (lane 3), indicating that these deleted sequences did not include functional sequences for SRSF9 in Caspase-2 AS. Collectively, deleted intron 9, exon 8, exon 10 and exon 9 in the deletion mutants above were not required for SRSF9 function. 

**Figure 3:**
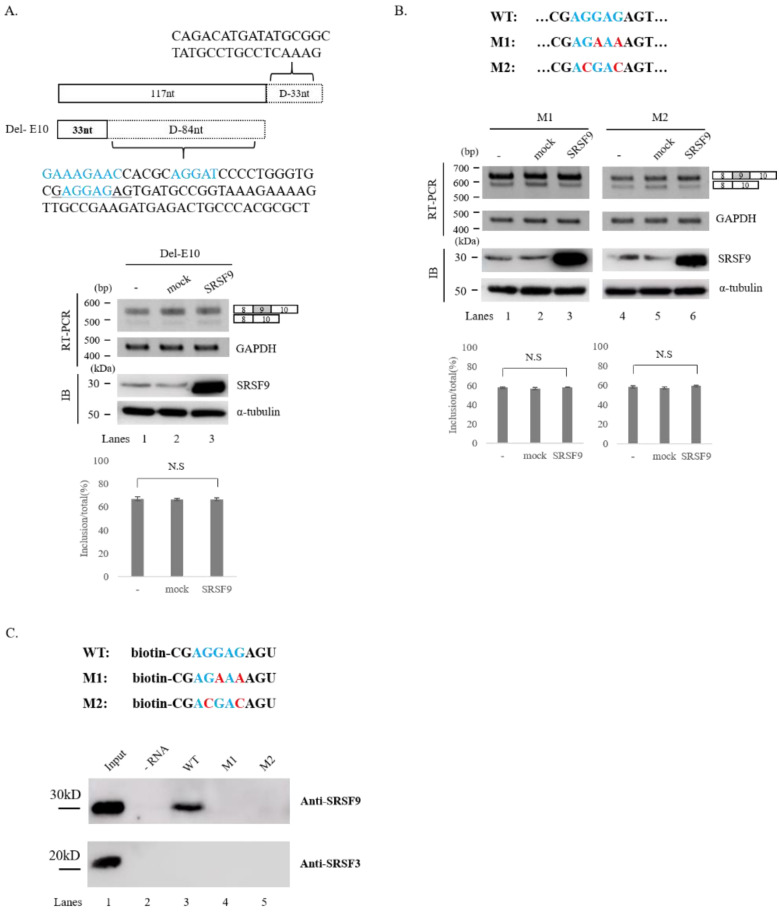
SRSF9 targets downstream exon to regulate AS of Caspase-2. (**A**)-(Upper) Schematic representation of D-10 minigene. Deleted sequences in the minigene are shown. D-10 was produced based on the deletion mutant of D9-2. (Middle) RT-PCR analysis of D-10 minigene in untreated, empty vector treated and SRSF9 expression plasmids treated HeLa cells. (Lower) Statistical differences are shown. (**B**)-(Upper) Sequences of wild type, M1 and M2 minigenes are shown.(Middle) RT-PCR analysis of M1 and M2 minigenes untreated, empty vector treated and SRSF9 expression plasmids treated HeLa cells are shown. (Lower) Statistical differences of RT-PCR analysis are shown. (**C**)-(Upper) Sequences of biotin-labeled oligonucleotides (wild type, M1 and M2) are shown. (Lower) Results of RNA-pulldown and immunoblotting for wild type, M1 and M2 with anti-SRSF9 and anti-SRSF3 antibodies are shown.

As we could not observe functions disruption of SRSF9 in the deletion of cassette exon, intron 9, we turned our attention to exon 10. We noticed that this exon contained several potential SRSF9 binding motifs or clusters (blue) as predicted with tools of SpliceAid2 and RBPmap (Figure 3A, Upper) [48,49]. To ask whether these motifs were required for SRSF9 function, we further deleted downstream 84 nt comprising all these motifs from D9-2 mutant, which produced exon 9 included isoform predominantly (Figure 2B, lane 1) to make it easier to observe the increase of skipped isoform (D-10) (Figure 3A, Upper). Importantly, SRSF9 function on Caspase-2 AS was completely disrupted in this mutant (lane 3) (Figure 3A, Lower). Thus, the deleted RNA should include functional sequences for SRSF9 in AS of Caspase-2. To further narrow down a specific functional motif of SRSF9, we produced two mutant minigenes in which AGGAG sequence (underlined) (Figure 3A, Upper) was substituted to AGAAA (M1) ACGAC (M2) (Figure 3B, Upper). As shown in Figure 3B (Lower), effects of SRSF9 on Caspase-2 AS were also completely hindered in both M1 and M2 mutants (lane 3 and 6). Therefore, we can conclude that SRSF9 functionally targets AGGAG sequence in exon 10 to regulate AS of Caspase-2 pre-mRNA.

### 3.3. SRSF9 Interacts with the Functional Target Sequence

As we found that AGGAG sequence in exon 10 targeted SRSF9 function in AS of Caspase-2, we next asked whether SRSF9 protein could interact with this sequence. To address this point, we synthesized 5′ biotin-labeled RNA of this sequence (WT) and two mutant sequences that resulted in functional disruption in Figure 3B (M1 and M2) (Figure 3C, Upper). We then performed RNA-pulldown analysis with streptavidin followed by immunoblotting with anti-SRSF9 antibody. Consistent with functional analysis, SRSF9 could interact with AGGAG sequence (WT) (lane 3), but not M1 or M2 sequences (Figure 3C, lanes 4 and 5). Combining functional analysis and physical interaction assay results, we conclude that SRSF9 functionally and physically can target the AGGAG sequence in exon 10.

### 3.4. SRSF9 Globally Affects AS of Apoptosis-Related Genes

**Figure 4:**
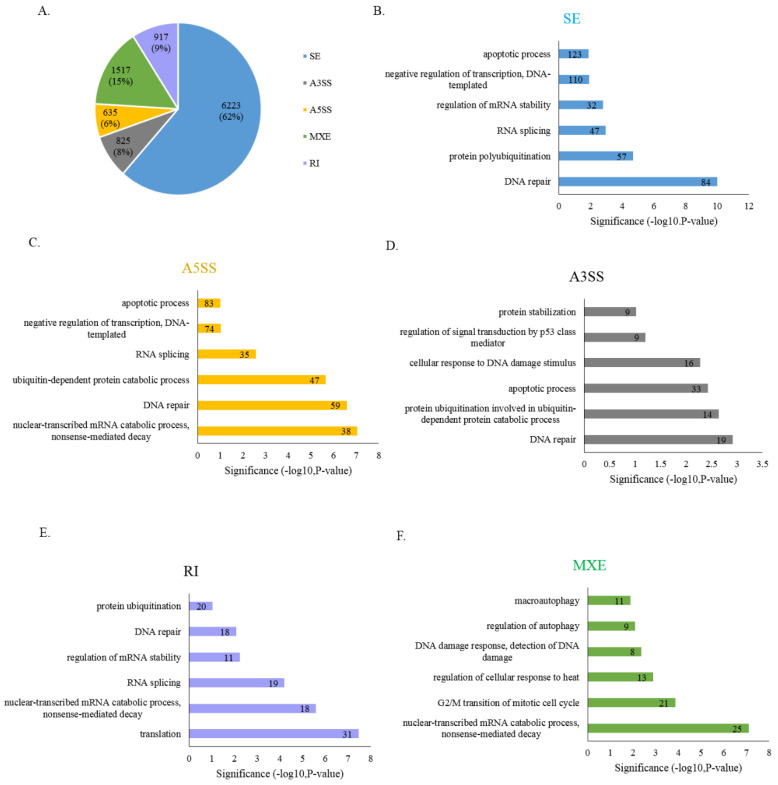
SRSF9 globally affects AS of apoptosis-related genes. (**A**). Regulated AS (SE, A5SS, A3SS, RI and MXE) events in SRSF9 KD cells with change in Percent Spliced In [ΔPSI] > 10 and *p* < 0.05. (**B**). GO analysis of genes enriched in SE events. (**C**). GO analysis of genes enriched in A5SS events. (**D**). GO analysis of genes enriched in A3SS events. (**E**). GO analysis of genes enriched in RI events. (**F**). GO analysis of genes enriched in MXE events.

Our results revealed that SRSF9 could regulate apoptosis related Caspase-2 AS. We further asked whether SRSF9 could regulate other apoptosis-related AS events. To this aim, we obtained RNA-seq results of SRSF9 KD compared to those of untreated HeLa cells available at NCBI Gene Expression Omnibus (GEO) (GSM1826780) [50]. We used rMATs algorithm [45] to analyze RNA-seq results and found that numerous AS events were altered by SRSF9 KD. As shown in Figure 4A, we observed that 6223 skipped exon (SE), 635 alternative 5′ splice-site selection (A5SS), 825 alternative 3′ splice-site selection (A3SS), 917 retained introns (RI) and 1517 mutually exclusive exons (MXE) differed significantly (change in Percent Spliced In [ΔPSI] > 10, *p* < 0.05) (Figure 4A) (Supplementary Table S3). To obtain functional insight of SRSF9, we used GO analysis to identify functional categories enriched within the set of genes that contained altered AS events by SRSF9 KD. We observed that genes containing SRSF9-regulated AS were not only directly enriched in apoptosis functions (Supplementary Table S4), but also enriched in functions linked to apoptosis, such as transcription, mRNA stability, RNA splicing, protein polyubiquitination, DNA repair translation, mitotic cell cycle and macroautophagy (Figure 4B–F). These results suggest that SRSF9 can globally regulate AS of apoptosis regulated exons. RT-PCR validation showed that AS of *Inhibitor of nuclear factor kappa B kinase regulatory subunit gamma* (*IKBKG*), *Peroxiredoxin 5* (*PRDX5*), *Galectin 1* (*LGALS1*) and *Cyclin dependent kinase 11B* (*CDK11B*) were significantly regulated by KD of SRSF9 (Supplementary Figure S3).

The fact that SRSF9 regulate AS of Caspase-2 and RNA-seq results imply a possibility that SRSF9 might regulate apoptosis. To address this possibility, we applied Annexin V/PI staining to assess the ratio of apoptotic cells. As shown in Supplementary Figure S4, while SRSF9-shRNA treated HeLa cells showed decreased apoptosis (7.14%) compared with NS-shRNA treated cells (23.6%), suggesting that SRSF9 regulate apoptosis.

## 4. Discussion

Apoptosis is a programed cell death occurring in normal cell turnover for proper development [12]. The importance of AS in apoptosis has been shown based on the fact that genes related to apoptotic regulation often produces oppositely acting protein variants to activate or inhibit apoptosis [51]. Similar to many genes involved in apoptosis, Caspase-2 generates two mRNA isoforms by AS. These isoforms are translated into two protein isoforms with opposite functions in apoptosis. Despite great results previously obtained, our understanding of molecular mechanisms by which Caspase-2 is splicing is still lacking. Our group has previously identified SRSF3 as a regulatory protein of Caspase-2 AS [30]. Continuing our previous work, in this manuscript, we found a new regulatory protein SRSF9 in the regulation of Caspase-2 AS. We observe a significant increase of cassette exon inclusion by SRSF9 KD and a significant decrease of skipped isoform. While SRSF3 functionally targets exon 8 [30], we show here that SRSF9 targets exon 10 and physically interacts with the target sequence. GO Analysis of RNA-seq demonstrated that SRSF9 had widespread effects on apoptosis related AS.

In addition to our finding that SRSF3 stimulates cassette exon splicing of Caspase-2 [30], AS of Caspase-2 has been shown to be regulated by other proteins. SRSF1, SRSF2, PTP, RBM5 hnRNP A1 [29,31,33]. Multiple regulatory sequence elements of Caspase-2 AS have been demonstrated. While regulatory elements located in intron 9 have been demonstrated to provide binding motifs for PTB and RBM5, sequences in exon 8 have also provided regulatory elements for SRSF3 [27,30,33]. We showed here that exon 10 also include a regulatory sequences for SRSF9. Whether these multiple regulatory sequences and regulatory proteins have combinatory or synergistic effects still need to be determined.

Caspase-2 pre-mRNA contains multiple potential binding sequences for SRSF9 as predicted using SpliceAid2 and RBPmap program [48,49]. However, our deletion mutagenesis analysis demonstrate that many potential sequences did not play important role in AS of Caspase-2. Interestingly, the RNA sequences located at exon 10 and 63 nt downstream of 3′ splice-site, but not the sequences of intron 9 or cassette exon, were important for SRSF9 function and furthermore, provided binding sequences for SRSF9. How the functional targets are selected still need to be studied. It has been found that SRSF9 interacts with Nop30, Y-box protein-1 [52,53]. Therefore, one possibility of the target sequence selection of SRSF9 might be through a protein complex of SRSF9 by which the target sequence but no other potential binding sequences are selected.

SRSF9 is closely related to SRSF1 (sharing ~74% amino acids). It comprises an unusually short RS domain [40]. However, the role of SRSF9 is not well understood as SRSF1. Our bioinformatical AS analysis using rMATs with RNA-seq from database demonstrate that SRSF9 has global impact on various AS events including SE, A3SS, A5SS, MXE and RI. Thus, similar to SRSF1, SRSF9 also have powerful effects on AS. SRSF9 was identified in subtractive protein analysis of Fas-induced apoptotic and non-apoptotic cells, implicating its roles in apoptosis [54]. SRSF9 has been shown to stimulate downstream 5′ splice-site of anti-apoptotic isoform of Bcl-x, a gene that can produce both pro-apoptotic and anti-apoptotic mRNA isoforms [39]. Here, we show that SRSF9 KD generates more anti-apoptotic cassette exon included isoform. RNA-seq demonstrate that, in addition to Caspase-2 AS, SRSF KD also regulates AS of other genes directly enriched in apoptosis, or indirectly enriched in apoptosis such as RNA splicing, DNA repair, protein ubiquitination and cell cycle. These results suggest the possibility that SRSF9 directly or indirectly regulates apoptosis signals.

## Data Availability

Not applicable.

## Supplementary Materials

The following are available online at https://www.mdpi.com/2073-4409/10/3/679/s1, Table S1: Primers used for RT-PCR analysis, Table S2: Predicted RNA binding motifs with RBPmap and SpliceAid 2, Table S3: List of GO analysis for genes associated with altered AS in SRSF9 KD, Table S4: List of SRSF9 KD affected AS-related genes, Figure S1: Potential SRSF9 binding locations in Caspase-2 pre-mRNA, Figure S2: Casp-2 exon 9 AS and SRSF9 expression levels correlate among different human tissues. Correlation analysis of Casp-2 exon 9 AS (PSI; Percent Spliced-In) and SRSF9 expression levels (cRPKM) in the indicated human tissues. Linear regression, Pearson correlation coefficient (r) and the calculated *p*-value are shown, Figure S3: RT-PCR validation of AS in *IKBKG*, *PRDX5*, *LGALS1* and *CDK11B* genes, Figure S4: Annexin V/PI staining of NS- or SRSF9-shRNA treated HeLa cells are shown. Red boxes indicate Annexin V(+) and PI(-) cells, the population of apoptotic cells, Figure S5: Densitometry analysis of immunoblotting in Figure 1, Figure 2 and Figure 3.
