# Supplementary material for: SRSF9 Regulates Cassette Exon Splicing of Caspase-2 by Interacting with Its Downstream Exon
Source: Cells. 2021 Mar 19;10(3):679. doi: 10.3390/cells10030679 (PMC8003524; doi:10.3390/cells10030679)
Supplement: Supplementary file 1 [file cells-10-00679-s001.zip › supplementary figures.pptx]

## Slide 1
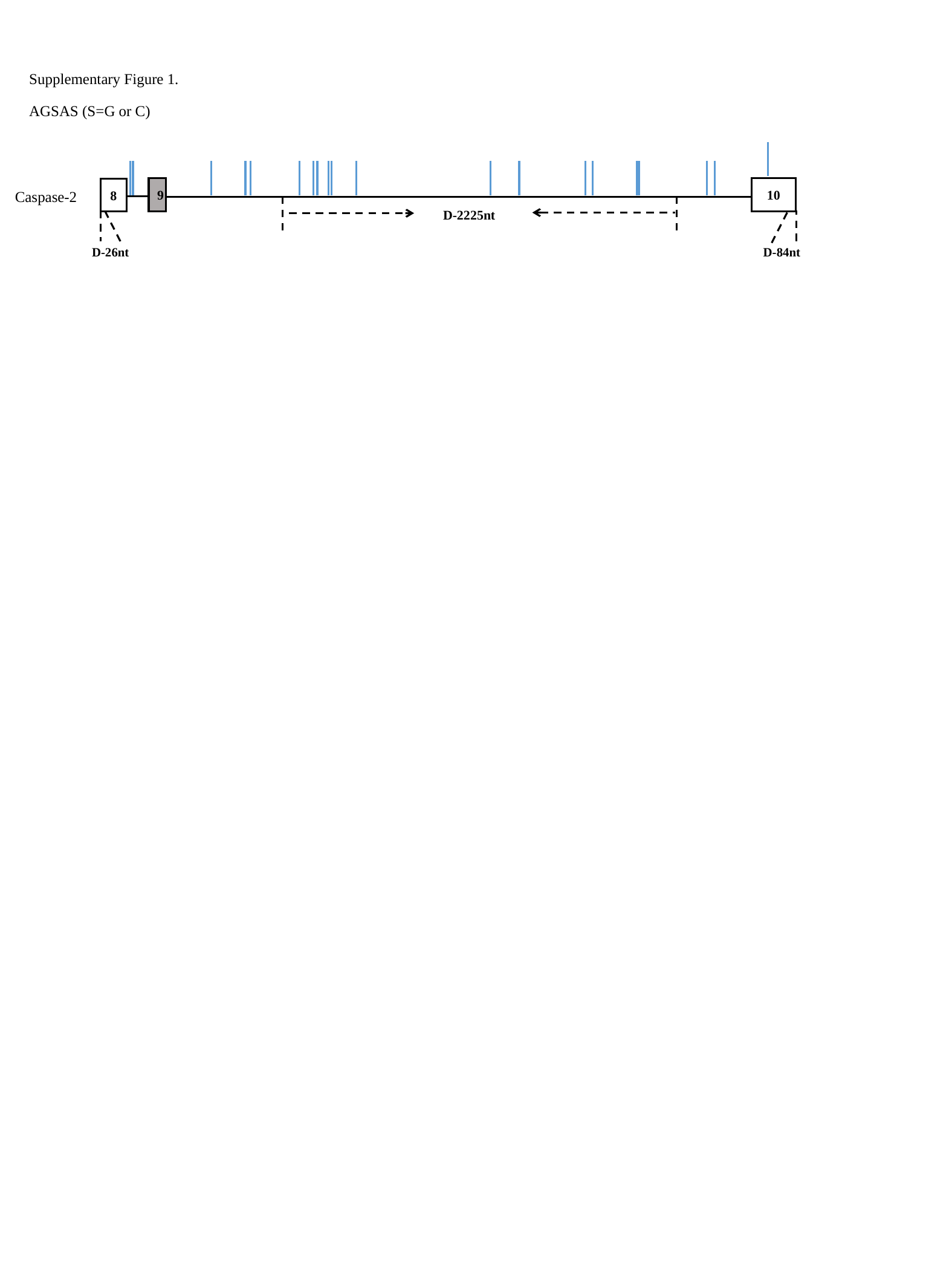

Supplementary Figure 1.
AGSAS (S=G or C)
9
10
8
Caspase-2
D-2225nt
D-26nt
D-84nt

## Slide 2
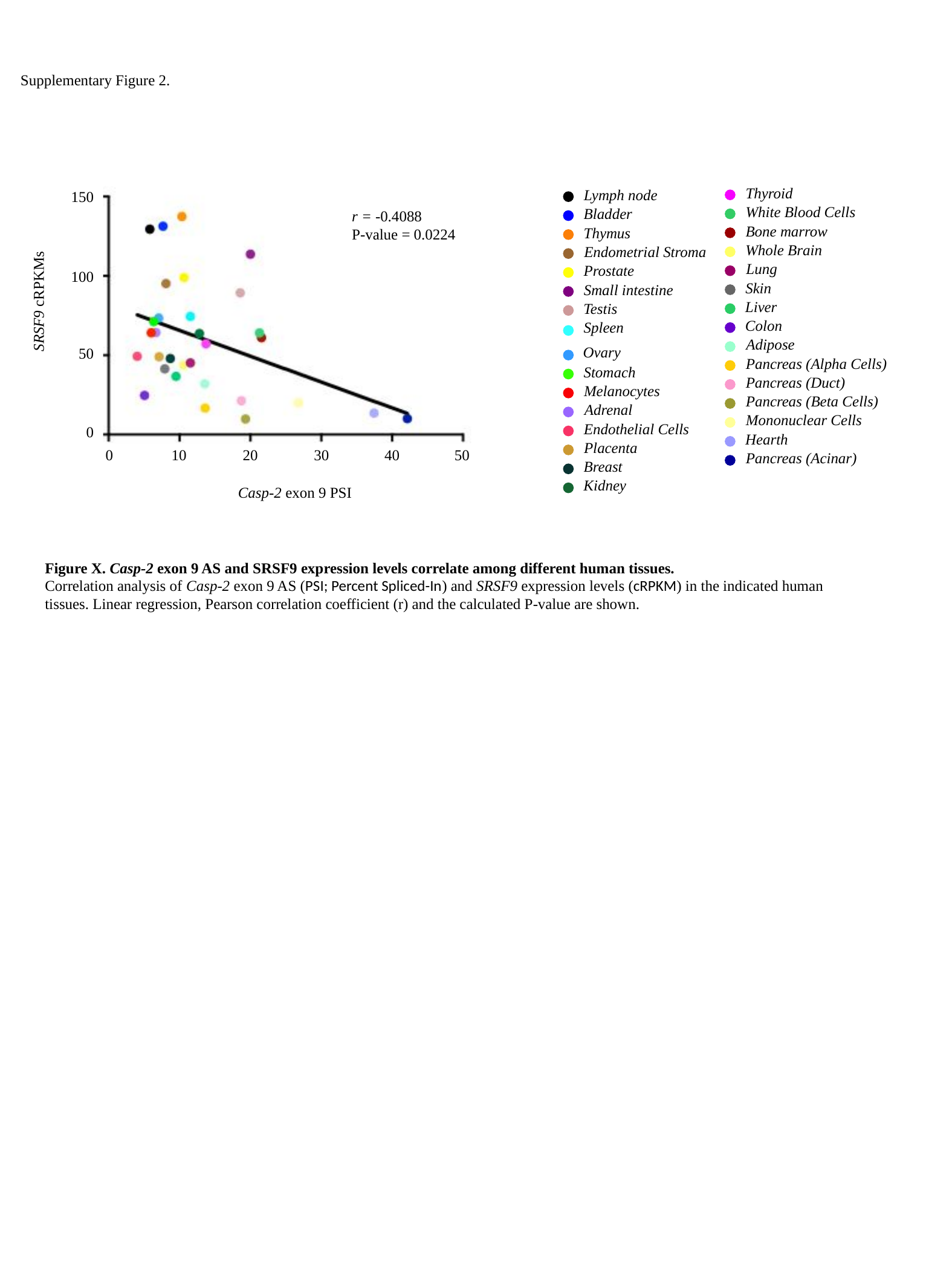

Supplementary Figure 2.
Thyroid
Lymph node
150
White Blood Cells
Bladder
r = -0.4088
P-value = 0.0224
Bone marrow
Thymus
Whole Brain
Endometrial Stroma
Lung
Prostate
100
Skin
Small intestine
SRSF9 cRPKMs
Liver
Testis
Colon
Spleen
Adipose
Ovary
50
Pancreas (Alpha Cells)
Stomach
Pancreas (Duct)
Melanocytes
Pancreas (Beta Cells)
Adrenal
Mononuclear Cells
Endothelial Cells
0
Hearth
Placenta
0
10
20
30
40
50
Pancreas (Acinar)
Breast
Kidney
Casp-2 exon 9 PSI
Figure X. Casp-2 exon 9 AS and SRSF9 expression levels correlate among different human tissues.
Correlation analysis of Casp-2 exon 9 AS (PSI; Percent Spliced-In) and SRSF9 expression levels (cRPKM) in the indicated human tissues. Linear regression, Pearson correlation coefficient (r) and the calculated P-value are shown.

## Slide 3
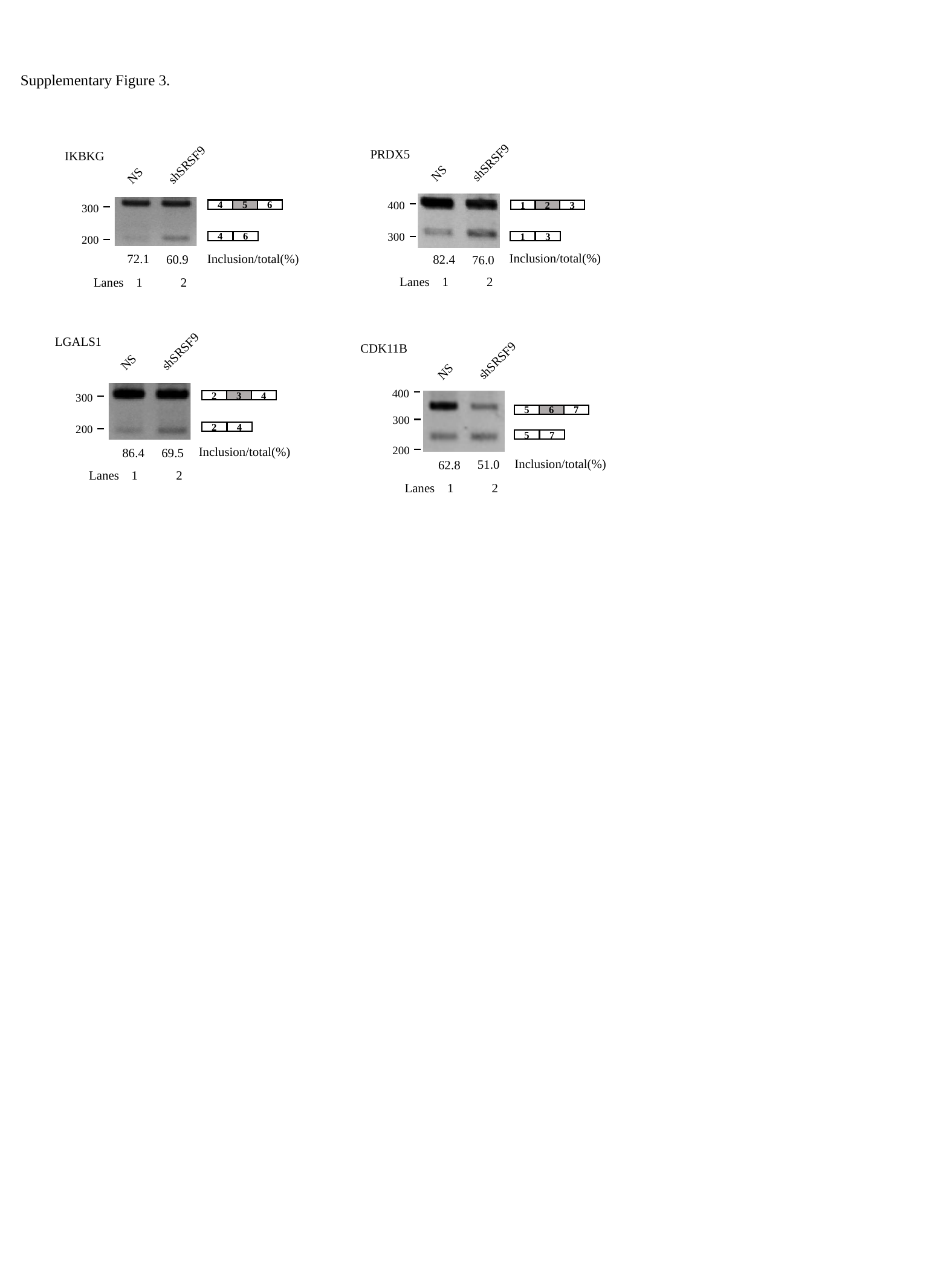

Supplementary Figure 3.
PRDX5
shSRSF9
NS
400
1
2
3
300
1
3
Inclusion/total(%)
82.4
76.0
Lanes 1 2
IKBKG
shSRSF9
NS
300
4
5
6
200
4
6
72.1
Inclusion/total(%)
60.9
Lanes 1 2
LGALS1
shSRSF9
NS
300
2
3
4
200
2
4
Inclusion/total(%)
86.4
69.5
Lanes 1 2
CDK11B
shSRSF9
NS
400
5
6
7
300
5
7
200
Inclusion/total(%)
51.0
62.8
Lanes 1 2

## Slide 4
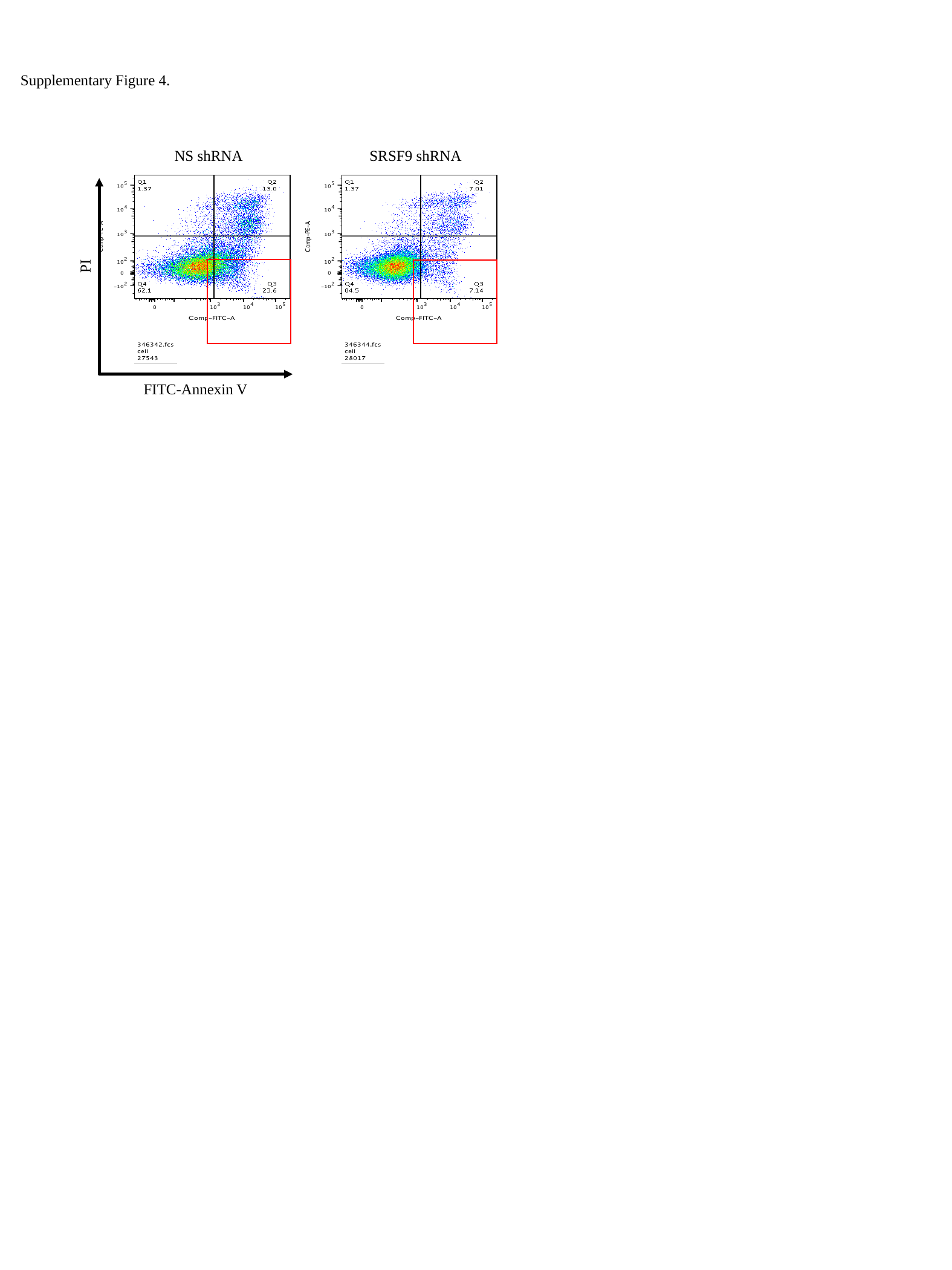

Supplementary Figure 4.
NS shRNA
SRSF9 shRNA
PI
FITC-Annexin V

## Slide 5
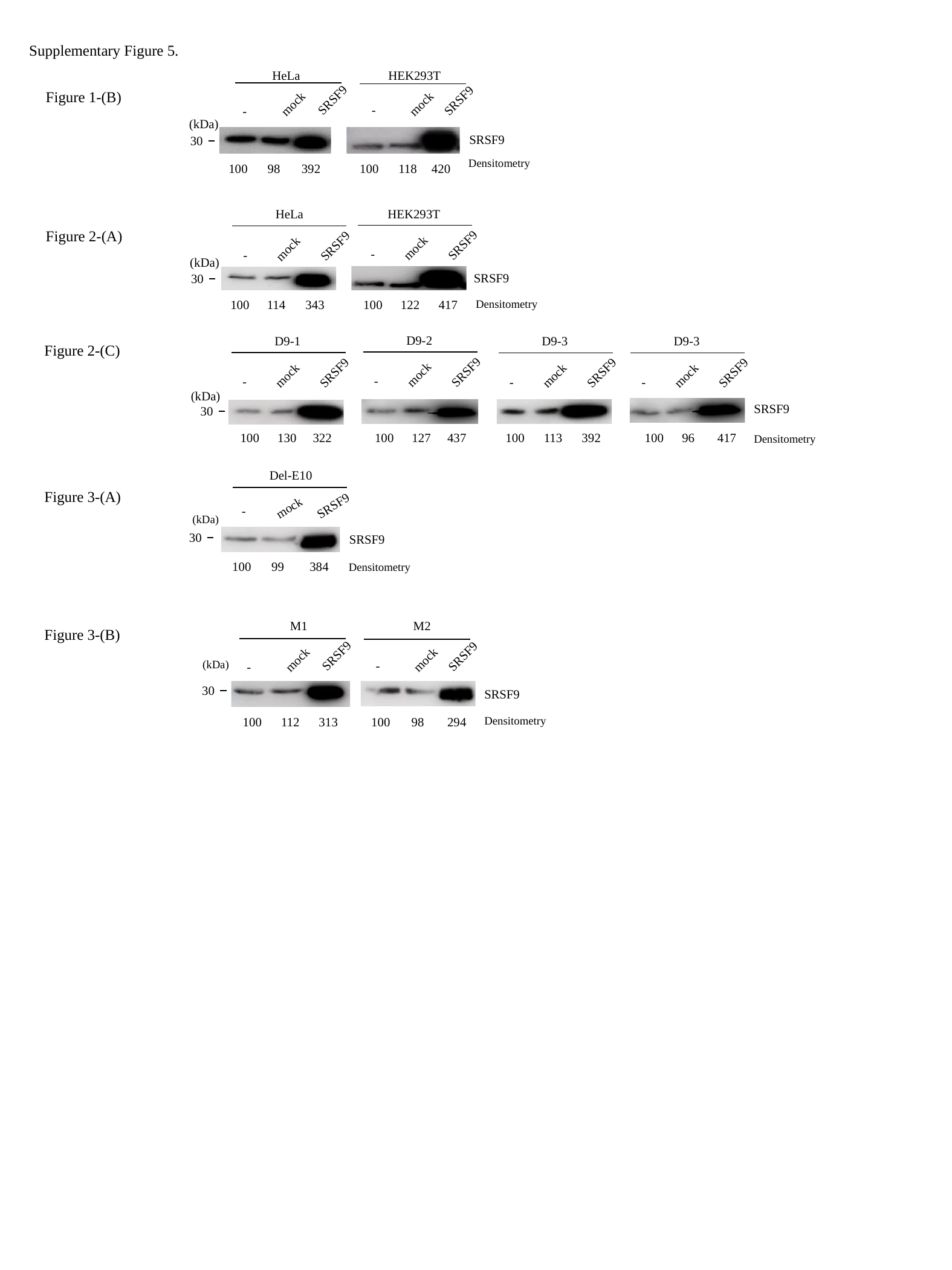

Supplementary Figure 5.
HEK293T
HeLa
Figure 1-(B)
SRSF9
SRSF9
mock
mock
-
-
(kDa)
SRSF9
30
Densitometry
100
98
392
100
118
420
HEK293T
HeLa
Figure 2-(A)
SRSF9
SRSF9
mock
mock
-
-
(kDa)
SRSF9
30
100
114
343
100
122
417
Densitometry
D9-2
D9-1
D9-3
D9-3
Figure 2-(C)
SRSF9
SRSF9
SRSF9
SRSF9
mock
mock
mock
mock
-
-
-
-
(kDa)
SRSF9
30
100
130
322
100
127
437
100
113
392
100
96
417
Densitometry
Del-E10
Figure 3-(A)
SRSF9
mock
-
(kDa)
30
SRSF9
100
99
384
Densitometry
M1
M2
Figure 3-(B)
SRSF9
SRSF9
mock
mock
(kDa)
-
-
30
SRSF9
Densitometry
100
112
313
100
98
294
